# Toxigenic *Vibrio cholerae* O1 in Water and Seafood, Haiti

**DOI:** 10.3201/eid1711.110748

**Published:** 2011-11

**Authors:** Vincent R. Hill, Nicole Cohen, Amy M. Kahler, Jessica L. Jones, Cheryl A. Bopp, Nina Marano, Cheryl L. Tarr, Nancy M. Garrett, Jacques Boncy, Ariel Henry, Gerardo A. Gómez, Michael Wellman, Maurice Curtis, Molly M. Freeman, Maryann Turnsek, Ronald A. Benner, Georges Dahourou, David Espey, Angelo DePaola, Jordan W. Tappero, Tom Handzel, Robert V. Tauxe

**Affiliations:** Centers for Disease Control and Prevention, Atlanta, Georgia, USA (V.R. Hill, N. Cohen, A.M. Kahler, C.A. Bopp, N. Marano, C.L. Tarr, N.M. Garrett, G.A. Gómez, M. Wellman, M. Curtis, M.M. Freeman, M. Turnsek, D. Espey, J.W. Tappero, T. Handzel, R.V. Tauxe); Food and Drug Administration, Dauphin Island, Alabama, USA (J.L. Jones, R.A. Benner, A. DePaola); Haitian Ministry of Public Health and Population, Port-au-Prince, Haiti (J. Boncy, A. Henry); Centers for Disease Control and Prevention, Port-au-Prince (G. Dahourou)

**Keywords:** Vibrio cholerae, cholera, drinking water, seafood safety, ultrafiltration

## Abstract

During the 2010 cholera outbreak in Haiti, water and seafood samples were collected to detect *Vibrio cholerae*. The outbreak strain of toxigenic *V. cholerae* O1 serotype Ogawa was isolated from freshwater and seafood samples. The cholera toxin gene was detected in harbor water samples.

Epidemic cholera is caused by toxigenic strains of *Vibrio cholerae* serogroups O1 and O139, which spread most often through water contaminated with the bacterium (*1*). Cholera can also be transmitted by eating contaminated foods, including seafood (*2*). Like other *V. cholerae* strains, which are autochthonous in riverine, estuarine, and coastal ecosystems, these strains may persist in the environment (*3*). An outbreak of cholera began in Haiti’s Artibonite Department in October 2010 and rapidly spread across all 10 Haitian departments and Port-au-Prince. Initial investigations indicated that drinking untreated water was the principal risk factor for infection (*4*). The ongoing risk posed to the Haitian population through contaminated water raised concern that cholera could also be introduced to other countries through transfer of *V. cholerae* by ship ballast water, contaminated seafood, or both. To better characterize the contamination of untreated surface water and seafood and to evaluate the risk for *V. cholerae* transfer from contaminated water in Haitian ports, the US Centers for Disease Control and Prevention (CDC) and the US Food and Drug Administration (FDA) collaborated with the Haitian ministries of health, agriculture, and environment to document the presence of *V. cholerae* in Haitian freshwater resources and harbors.

## The Study

In October and November 2010, water and seafood samples were collected from 2 cholera-affected communities in Haiti and tested for *V. cholerae*. Eight freshwater and 6 marine water samples were collected from 13 sites in Artibonite and Ouest Departments (Figure). Freshwater samples were collected from rivers, including the Artibonite River, and irrigation canals. Dead-end ultrafiltration, a newly developed technique that has been used to recover diverse microbes from large-volume water samples (*5*), was used to collect water samples (8–30 L each) at the freshwater sites and 3 of the marine water sites (HWS-11, -13, and -18). At the Haiti National Public Health Laboratory (LNSP), bacteria were recovered from ultrafilters by back flushing with a surfactant solution, and the solution then was added to an equal volume of 2× strength alkaline peptone water (APW). Grab samples (1 L) were collected at 3 harbor sites (HWS-15, -16, and -17) and on arrival at LNSP, they were split into two 500-mL portions for separate testing by CDC at LNSP or for shipment in chilled coolers to FDA (Dauphin Island, AL, USA). At LNSP, all water and ultrafilter back flush samples were incubated in APW at 37°C for 6 h (*6*). After APW enrichment, the culture broth was streaked onto thiosulfate citrate bile salts (TCBS) agar (Remel, Lenexa, KS, USA) and incubated overnight at 37°C. For each sample, up to 10 colonies suspected of being positive were picked from TCBS agar and grown on nonselective media for multiplex PCR testing (*7*).

**Figure:**
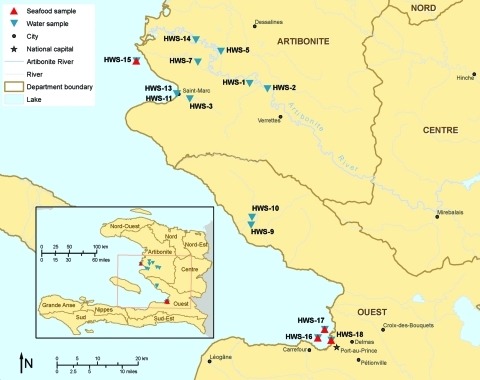
Locations where water and seafood samples were obtained, Haiti, October–November 2010. HWS, harbor water sample.

Nine seafood samples were collected along the coast: 5 between Saint-Marc port and Grand Saline (1 site) and 4 from Port-au-Prince port (3 sites) (Figure). Seafood samples were obtained as convenience samples from local fishermen on the water, placed in Ziploc (SC Johnson, Racine, WI, USA) bags, and sent in chilled coolers to FDA, along with 500-mL grab samples of marine water. After enrichment, APW cultures from seafood and water grab samples were tested at FDA by using a real-time PCR specific to the cholera toxin gene (*ctxA*) of *V. cholerae* (*8*). If the APW culture was positive for the *ctxA* gene, then isolates were obtained by streaking onto TCBS agar as described for water samples.

Identification and characterization of suspected *V. cholerae* isolates were performed at CDC. Colonies or sweeps of growth typical of *V. cholerae* were selected from TCBS plates and tested by multiplex PCR for *ctxA*, *tcpA*_El Tor_, *tcpA*_Classical_, *ompW*, and *toxR* genes (*9**–**11*). Colonies positive by PCR for *ctxA* or other *V. cholerae* markers were tested for agglutination in serogroup O1 antiserum and, if positive, also in Inaba and Ogawa serotype antisera. *V. cholerae* isolates were subtyped according to the PulseNet standardized pulsed-field gel electrophoresis (PFGE) protocol, using primary and secondary restriction enzymes *Sfi*I and *Not*I, respectively (*12*). The cholera toxin gene, *ctxAB*, and *tcpA* gene regions were amplified by PCR and sequenced (*13**,**14*).

*V. cholerae* O1, serotype Ogawa, *ctxA*-positive strains were isolated from 2 irrigation canals north of Port-au-Prince in Ouest Department (Table 1). Both of these canals were used for drinking water by the local population, and communities near the canals were heavily affected by the outbreak. *V. cholerae* O1 Ogawa *ctxA*-positive strains were isolated from 1 mixed seafood sample (sample 7, containing multiple vertebrate fish and 1 crab) and 1 bivalve sample (sample 8, containing multiple species) that were obtained from fishermen at 2 different locations in the Port-au-Prince port (Table 2). All *ctxA*-positive *V. cholerae* isolates were indistinguishable from the outbreak strain by PFGE with both enzymes (pattern combination KZGS12.0088/KZGN11.0092) (*15*). Sequence analysis for the toxigenic *V. cholerae* isolates provided additional evidence that the isolates from these samples matched the isolates from humans infected with the outbreak strains. The *tcpA* sequence of the freshwater and human isolates from Haiti matched that of CIRS 101, an altered El Tor strain from Bangladesh, and the *ctxAB* sequences matched the sequences from strains isolated in 2007 during an outbreak in Orissa, India (*15*). The *ctxAB* and *tcpA* sequences differed by 1 nt polymorphism from prototypical classical and El Tor alleles, respectively. These isolates were recovered from 30-L freshwater samples having turbidities of 11 and 16 nephelometric turbidity units, which were among those with the lowest turbidity collected during this investigation. All *V. cholerae* non-O1 *ctxA*-negative strains possessed unique PFGE patterns distinct from the outbreak pattern. In addition to samples from which toxigenic *V. cholerae* was isolated, real-time PCR testing by FDA detected the *ctxA* gene in APW culture broths for 3 seawater samples and 3 other seafood samples.

**Table 1:** Results of water sampling for *Vibrio cholerae*, Haiti, October–November 2010*

Sample no.	Sample location	Sample types	Collection date	Volume sampled, L	Turbidity, NTU	APW broth real-time PCR result	Characterization of *V. cholerae* isolates
HWS-1	Liancourt River	UF	Oct 29	7.7	150	ND	No isolate obtained
HWS-2	Artibonite River	UF	Oct 29	16	250	ND	No isolate obtained
HWS-3	Obya River	UF	Oct 30	35	31	ND	Non-O1, non-O139, *ctxA* negative
HWS-5	Sipa Canal	UF	Oct 30	32	88	ND	Non-O1, non-O139, *ctxA* negative
HWS-7	Brown Root River	UF	Oct 30	21	11	ND	Non-O1, non-O139, *ctxA* negative
HWS-9	Freshwater canal (canal 2)	UF	Nov 2	30	16	ND	*V. cholerae* isolate matched outbreak strain†
HWS-10	Freshwater canal (canal 1)	UF	Nov 2	30	11	ND	*V. cholerae* isolate matched outbreak strain†
HWS-11	Saint Marc port marine water	UF	Nov 9	30	ND	ND	No isolate obtained
HWS-13	Saint Marc port marine water	UF	Nov 9	30	ND	ND	No isolate obtained
HWS-14	Grand Saline canal	UF	Nov 10	20	170	ND	Non-O1, non-O139, *ctxA* negative
HWS-15	Saint Marc/Grand Saline port marine water	Grab	Nov 9	1	ND	*ctxA* detected	No isolate obtained
HWS-16	Port-au-Prince port, site 1 marine water	Grab	Nov 11	1	ND	*ctxA* detected	No isolate obtained
HWS-17	Port-au-Prince port, site 2 marine water	Grab	Nov 11	1	ND	*ctxA* detected	No isolate obtained
HWS-18	Port-au-Prince port, site 3 marine water	UF	Nov 11	28	ND	ND	No isolate obtained

*NTU, nephelometric turbidity units; APW, alkaline peptone water; HWS, harbor water sample; UF, ultrafiltration; ND, no data. †*V. cholerae* serogroup O1, serotype Ogawa, biotype El Tor, *ctxA* positive, pulsed-field gel electrophoresis–matched outbreak strain.

**Table 2:** Results of seafood sampling for *Vibrio cholerae*, Haiti, October–November 2010*

Sample no.	Sample location†	Seafood type	APW broth real-time PCR result	Characterization of *V. cholerae* isolates
1	Saint Marc/Grand Saline	Oysters	*ctx*A detected	No isolate obtained
2	Saint Marc/Grand Saline	Red mussels	No *ctx*A detected	No isolate obtained
3	Saint Marc/Grand Saline	Queen conch	*ctx*A detected	No isolate obtained
4	Saint Marc/Grand Saline	Conch	No *ctx*A detected	No isolate obtained
5	Saint Marc/Grand Saline	Clams	No *ctx*A detected	No isolate obtained
6	Port-au-Prince, site 1	Octopus	No *ctx*A detected	No isolate obtained
6	Port-au-Prince, site 1	Clams	*ctx*A detected	No isolate obtained
6	Port-au-Prince, site 1	Assorted gastropods	*ctx*A detected	No isolate obtained
6	Port-au-Prince, site 1	Assorted bivalves	*ctx*A detected	No isolate obtained
7	Port-au-Prince, site 2	Fish and crab combined sample	*ctx*A detected	Isolate matched outbreak strain‡
8	Port-au-Prince, site 3	Assorted bivalves	*ctx*A detected	Isolate matched outbreak strain‡
9	Port-au-Prince, site 3	Mussels	No *ctx*A detected	No isolate obtained

*ID, identification; APW, alkaline peptone water. †Samples 1–5, sample 6, sample 7, and samples 8 and 9 were obtained at the same locations as water samples HWS-15, HWS-16, HWS-17, and HWS-18, respectively (Table 1; Figure). ‡*V. cholerae* serogroup O1, serotype Ogawa, biotype El Tor, *ctxA*-positive, pulsed-field gel electrophoresis–matched outbreak strain.

## Conclusions

Isolation of the outbreak strain in seafood samples from Port-au-Prince and detection of the *ctxA* gene in APW cultures of water and seafood samples from Port-au-Prince and Saint-Marc suggest that harbor waters were contaminated with toxigenic *V. cholerae* O1. This finding underscores the need for adherence to public health recommendations disseminated during the outbreak regarding making drinking water safe and cooking seafood thoroughly to prevent infection and conducting ship ballast water exchange to limit potential transfer of the organism to other harbors. We report recovery of *V. cholerae* O1 from large-volume water samples by use of ultrafiltration. Although *V. cholerae* O1 was not isolated from marine water samples, real-time PCR detection of *ctxA* in these samples provided additional evidence that harbor water samples were contaminated with toxigenic *V. cholerae*. Use of this real-time PCR method has provided analytical data that reflected the presence of viable *V. cholerae* in marine water samples (*8*). Further assessment by using high-volume filtration and seafood sampling may be useful for tracking the persistence of the strain in the Haitian environment in the future.
